# Association between serum adiponectin concentrations and chronic obstructive pulmonary disease: a meta-analysis

**DOI:** 10.1042/BSR20192234

**Published:** 2020-03-12

**Authors:** Yi-Hua Lin, Tian-Xiang Jiang, Su-Xian Hu, Yong-Hong Shi

**Affiliations:** 1 Department of Respiratory and Critical Care Medicine, the First Affiliated Hospital of Xiamen University, Xiamen, Fujian, China; 2 Outpatient Department, the First Affiliated Hospital of Xiamen University, Xiamen, Fujian, China

**Keywords:** adiponectin, Chronic obstructive pulmonary disease, meta-analysis

## Abstract

**Background:** Adiponectin has been implicated to play a role in the pathophysiology of chronic obstructive pulmonary disease (COPD). Many studies have assessed serum adiponectin concentrations in COPD patients. However, results from different reports were not consistent. To assess the association of serum adiponectin concentrations and COPD, a meta-analysis was performed.

**Methods:** PubMed, Embase, Web of Science and Cochrane Library were searched for eligible studies. Data were extracted, and then standard mean differences (SMDs) and 95% confidence intervals (CI) were calculated.

**Results:** Thirteen studies involving a total of 1131 cases and 689 controls were included in this meta-analysis. Combined data indicated that the serum adiponectin levels were higher in COPD patients than those in controls (SMD: 1.09, 95% CI [0.73–1.45], *P* < 0.001). In the subgroup analyses by disease period, there were similar results in stable COPD patients (SMD: 0.77, 95% CI [0.47–1.07], *p* <0.001; *I*^2^ = 83.9%, *P* < 0.001), AECOPD patients (SMD: 2.51, 95% CI [0.71–4.30], *P* = 0.006; *I*^2^ = 95.2%, *P* < 0.001) and mixed COPD patients (SMD: 1.21, 95% CI [0.67–1.75], *P* < 0.001). Furthermore, the serum adiponectin levels were higher in AECOPD patients than those in stable COPD patients (SMD: 1.06, 95% CI [0.13–1.99], *P* = 0.026).

**Conclusions:** This meta-analysis indicates that patients with COPD have higher serum adiponectin concentration than healthy controls.

## Introduction

Chronic obstructive pulmonary disease (COPD) is one of the major causes of mortality throughout the world [1]. The overall prevalence of spirometry-defined COPD was 8.6%, accounting for 99.9 million people with COPD in China [2]. More and more studies have shown that COPD is not only a respiratory inflammatory condition, but also one disease characterized by low-grade and chronic systemic inflammation with many extrapulmonary manifestations [3,4]. The systemic inflammation is associated with lung function, exercise capacity, degree of dyspnea, arterial oxygen tension, clinical outcome, risk of exacerbation, and all-cause and COPD-related mortalities [5]. Moreover, the systemic inflammation is associated with increased risk of major comorbidities in COPD, including lung cancer, cardiovascular disease, depression, cachexia, pneumonia, diabetes mellitus, skeletal muscle dysfunction and osteoporosis [6].

In recent years, adipose tissue has also been reported to interfere with the systemic inflammation in COPD patients. In addition to functioning as the energy storage site, the adipose tissue is an active producer of mediators involved in inflammation, and the so-called adipokines are examples [7]. Adiponectin is one of these adipokines and has important anti-inflammatory, anti-atherosclerotic and antiobesity effects. Both increased adiponectin levels in bronchoalveolar lavage fluid and increased adiponectin expression by airway epithelial cells were found in tobacco-induced COPD mice [8,9]. These animal studies have prompted attention to the dysregulation of adiponectin and the progression of COPD. In last decade, several reports have assessed serum adiponectin concentrations in COPD patients. However, results from different reports were not consistent. Besides, most of the studies undertaken to evaluate this potential relationship were in small scale, and therefore may not be able to clarify this issue due to insufficient statistical evidence. In order to better evaluate the relationship between serum adiponectin concentrations and COPD, a meta-analysis was conducted.

## Methods

### Literature sources and search strategy

Two investigators (Y.-H. Lin and T.-X. Jiang) independently searched PubMed, Embase, Web of Science and Cochrane Library up to November 26, 2019 to identify potentially relevant articles. Disagreements were resolved via discussion or adjudicated by a third author (Y.-H. Shi). The following search terms were used in Pubmed: (((((“Pulmonary Disease, Chronic Obstructive” [Mesh]) OR (((((((COPD [Title/Abstract]) OR Chronic Obstructive Pulmonary Disease [Title/Abstract]) OR COPD [Title/Abstract]) OR Chronic Obstructive Airway Disease [Title/Abstract]) OR Chronic Obstructive Lung Disease [Title/Abstract]) OR Chronic Airflow Obstructions [Title/Abstract]) OR Chronic Airflow Obstruction [Title/Abstract]))))))) AND ((adiponectin [Title/Abstract]) OR “Adiponectin” [Mesh]). There was no restriction on language. References of all selected articles were retrieved to identify other relevant studies.

### Inclusion and exclusion criteria

Inclusion criteria were as follows: (1) observational studies reporting the serum adiponectin levels both in healthy control group and the COPD group, including stable stage and acute exacerbation stage of COPD (AECOPD); (2) COPD patients who met the criteria of the American Thoracic Society or European Respiratory Society or Global Initiative for Chronic Obstructive Lung Disease (GOLD); (3) healthy controls with no COPD.

Exclusion criteria were as follows: (1) significant difference between the two groups for baseline age, gender, or body mass index (BMI); (2) no data to show the difference between the two groups for BMI; (3) patients with a history or diagnosis of asthma, allergy or other respiratory diseases other than COPD; (4) articles with no original data.

### Quality assessment

The Newcastle–Ottawa scale (NOS), which assesses the quality of non-randomized studies on the basis of selection of participants, comparability of groups, and exposure assessment, was used by two authors for evaluating the study quality independently. Disagreement was settled as described above.

### Data extraction

When the titles and abstracts met the inclusion criteria, full articles were searched and screened by two investigators to confirm eligibility independently (Y.-H Lin and T-X Jiang). Disagreements were resolved as described above. The following information was extracted: first author, year of publication, original country, sample size, age, gender, BMI, smoking status, source of controls, COPD definition, lung function of cases, methods of measuring adiponectin, mean value and standard deviation (SD) of serum adiponectin levels in both COPD patients and healthy subjects. If the included studies provided data of median and interquartile range, the data were transformed to mean and SD according to the method proposed by Hozo et al. [10]

### Statistical analysis

The data were analyzed using STATA (StataCorp. 2015. *Stata Statistical Software: Release 14.* College Station, TX: StataCorp LP.). Due to the various measuring methods with different units for adiponectin, standardized mean differences (SMDs) with 95% confidence intervals (CIs) were selected to combine statistics. Pooled SMD with 95% CI was calculated and *P* < 0.05 was recognized as statistical significance. Heterogeneity was checked by the *Q*-test and *I*^2^-statistics [11]. Meta-analysis was done using the fixed-effects model when *P*-value of the *Q*-test was > 0.10. Otherwise, the random-effects model was applied [12]. *I*^2^ values were used to quantify heterogeneity [13]. The leave-one-out sensitivity analysis was performed by extracting a single study each time to check the stability of the results. For the subgroup analyses, the study populations were stratified into three groups: stable COPD subgroup, AECOPD subgroup and mixed COPD subgroup. Further subgroup analyses were performed to evaluate the influence of main confounders. Publication bias was assessed using Egger’s linear regression test, Begg’s rank correlation test and funnel plots [14].

## Results

### Study inclusion and characteristics

**Figure 1:**
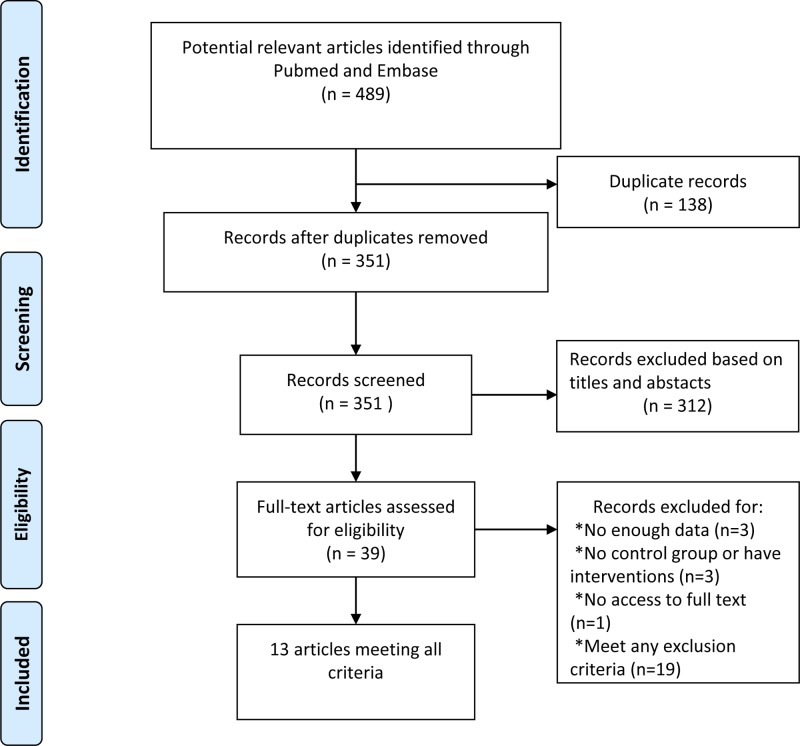
Flow of study indentification, inclusion and exclusion

The procedure for identifying and selecting eligible studies as showed in Figure 1. A total of 489 articles were retrieved after initial search. Among them, 138 duplicate records were removed, leaving 351 papers for screening. Three hundred and twelve articles were excluded based on titles and abstracts, and then twenty-six records were excluded after full-text review. Finally, 13 articles were included for this meta-analysis, containing 1131 COPD patients and 689 controls in total. The subjects of nine studies were stable COPD patients [15–23], three studies involved stable COPD patients and AECOPD patients [24–26], and one study involved mixed COPD patients [27]. In 12 studies [15–23,25–27], the control participants were matched for all the main confounders (BMI, age, gender) with the OSA patients. In one study [24], the information of participants’ age was not mentioned. The NOS scores of the 13 articles ranged from 7 to 9, indicating that the methodological quality was generally good. The main characters of all the including studies are presented in Table 1. The main confounders and adiponectin levels in the studies included in the meta-analysis are presented in Table 2.

**Table 1:** The basic characteristics of studies included in the meta-analysis

Study (First author, Year)	Country	Numbers		Disease period	GOLD stages of cases	NOS
		Cases	Controls			
Tomada, 2007	Japan	12	12	Stable COPD	No stage information	7
Kirdar, 2009a	Turkey	15	17	Stable COPD	GOLD stage I-IV	8
Kirdar, 2009b	Turkey	21	17	AECOPD	No stage information	8
Xie, 2010a	China	30	30	Stable COPD	No stage information	7
Xie, 2010b	China	30	30	AECOPD	No stage information	7
Gaki, 2011	Greece	222	132	Stable COPD	No stage information	7
Breyer, 2011	Netherlands	91	36	Stable COPD	GOLD stage Ⅱ II-IV	9
Breyer, 2012	Netherlands	186	133	Stable COPD	GOLD stage Ⅱ II-IV	9
Yoshikawa, 2012	Japan	30	41	Stable COPD	No stage information	7
Mohamed, 2013	Egypt	60	20	Mixed	No stage information	7
Liu, 2013a	China	19	35	Stable COPD	No stage information	7
Liu, 2013b	China	145	35	AECOPD	No stage information	7
Li, 2015	China	73	54	Stable COPD	No stage information	7
Zhang, 2016	China	50	39	Stable COPD	No stage information	8
Wu, 2016	China	73	40	Stable COPD	No stage information	7
Waschki, 2017	Germany	74	18	Stable COPD	GOLD stage I-IV	8

Abbreviations: AECOPD, acute exacerbation stage of chronic obstructive pulmonary disease; COPD, chronic obstructive pulmonary disease; GOLD, Global Initiative for Chronic Obstructive Lung Disease; NOS, Newcastle–Ottawa scale.

**Table 2:** The main confounders and adiponectin levels in the studies included in the meta-analysis

Study (first author, year)	Age (years)		Gender (male/female)		BMI (kg/m^2^)		Adiponectin levels	
	Cases	Controls	Cases	Controls	Cases	Controls	Cases	Controls
Tomada, 2007	71.60 ± 1.30	69.30 ± 1.60	12/0	12/0	23.80 ± 1.73	24.30 ± 2.08	11.90 ± 5.20 (μg/ml)	5.20 ± 2.43 (μg/ml)
Kirdar, 2009a	71.52 ± 9.90	65.71 ± 6.30	15/0	Matched	26.43 ± 3.80	26.70 ± 3.00	11.73 ± 2.60 (ng/ml)	7.58 ± 3.20 (ng/ml)
Kirdar, 2009b	67.70 ± 8.90	65.71 ± 6.30	21/0	Matched	24.32 ± 3.01	26.70 ± 3.00	18.29 ± 8.90 (ng/ml)	7.58 ± 3.20 (ng/ml)
Xie, 2010a	65.64 ± 2.55	65.60 ± 2.19	30/0	30/0	23.05 ± 0.98	23.43 ± 0.47	10.90 ± 4.09 (ng/ml)	5.86 ± 1.08 (ng/ml)
Xie, 2010b	64.21 ± 2.84	65.60 ± 2.19	30/0	30/0	22.73 ± 1.20	23.43 ± 0.47	18.02 ± 3.37 (ng/ml)	5.86 ± 1.08 (ng/ml)
Gaki, 2011	63.75 ± 3.76	59.50 ± 3.45	169/53	97/35	27.50 ± 4.00	28.00 ± 5.00	4.15 ± 2.98 (μg/ml)	4.37 ± 2.10 (μg/ml)
Breyer, 2011	62.25 ± 4.34	Matched	46/45	24/12	24.76 ± 6.63	Matched	10.29 ± 6.72 (μg/ml)	7.87 ± 3.24 (μg/ml)
Breyer, 2012	57.15 ± 3.17	57.50 ± 3.45	Matched	Matched	27.65 ± 6.32	29.00 ± 6.00	9.87 ± 4.93 (ng/ml)	8.02 ± 4.43 (ng/ml)
Yoshikawa, 2012	66.50 ± 2.92	67.5 ± 1.12	30/0	41/0	21.04 ± 2.65	21.59 ± 3.15	11.99 ± 5.14 (μg/ml)	7.42 ± 1.31 (μg/ml)
Mohamed, 2013	66.70 ± 6.90	62.72 ± 4.20	Matched	Matched	25.30 ± 3.00	26.40 ± 4.00	14.54 ± 7.60(μg/ml)	6.55 ± 0.30 (μg/ml)
Liu, 2013a	NM	NM	Matched	Matched	Matched	Matched	6.42 ± 1.35 (μg/ml)	5.21 ± 1.30 (μg/ml)
Liu, 2013b	NM	NM	Matched	Matched	Matched	Matched	6.92 ± 1.33 (μg/ml)	5.21 ± 1.30 (μg/ml)
Li, 2015	63.83 ± 9.97	61.22 ± 11.49	50/23	29/25	22.88 ± 2.78	23.73 ± 3.53	8.24 ± 4.86 (μg/ml)	5.46 ± 2.57 (μg/ml)
Zhang, 2016	64.14 ± 10.21	60.92 ± 9.62	30/20	23/16	22.56 ± 2.60	23.59 ± 3.61	7.81 ± 4.37 (μg/ml)	5.64 ± 2.69 (μg/ml)
Wu, 2016	61.29 ± 10.83	59.5 ± 11.43	51/22	22/18	22.83 ± 2.70	23.72 ± 3.60	7.76 ± 4.73 (μg/ml)	5.27 ± 2.37 (μg/ml)
Waschki, 2017	66.00 ± 6.60	65.90 ± 5.80	52/22	11/7	26.00 ± 5.30	25.70 ± 4.60	8.67 ± 4.11 (ng/ml)	6.20 ± 3.16 (ng/ml)

Abbreviations: BMI, body mass index; NM, not mentioned. Data are presented as mean ± standard deviation unless otherwise stated.

### Quantitative analysis

**Figure 2:**
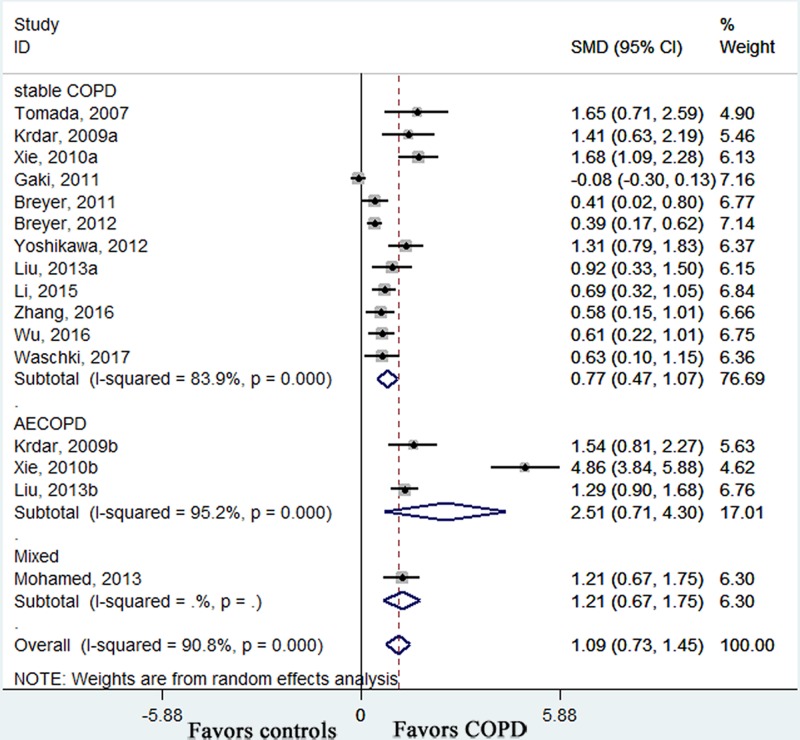
Forest plots of SMD with 95% CI for the circulating level of adiponectin in COPD patients compared with controls SMD: standardized mean difference; CI: confidence interval; COPD: chronic obstructive pulmonary disease; AECOPD: acute exacerbation stage of COPD.

**Figure 3:**
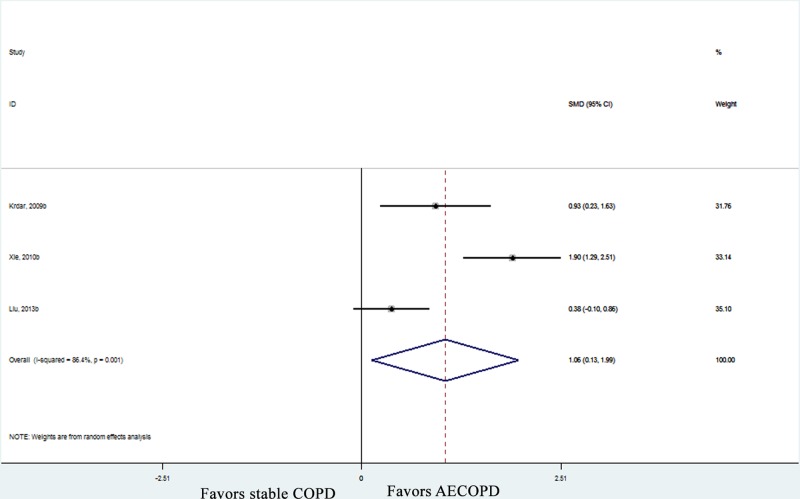
Forest plots of SMD with 95% CI for the circulating level of adiponectin in AECOPD patients compared with stable COPD patients SMD: standardized mean difference; CI: confidence interval; COPD: chronic obstructive pulmonary disease; AECOPD: acute exacerbation stage of COPD.

**Figure 4:**
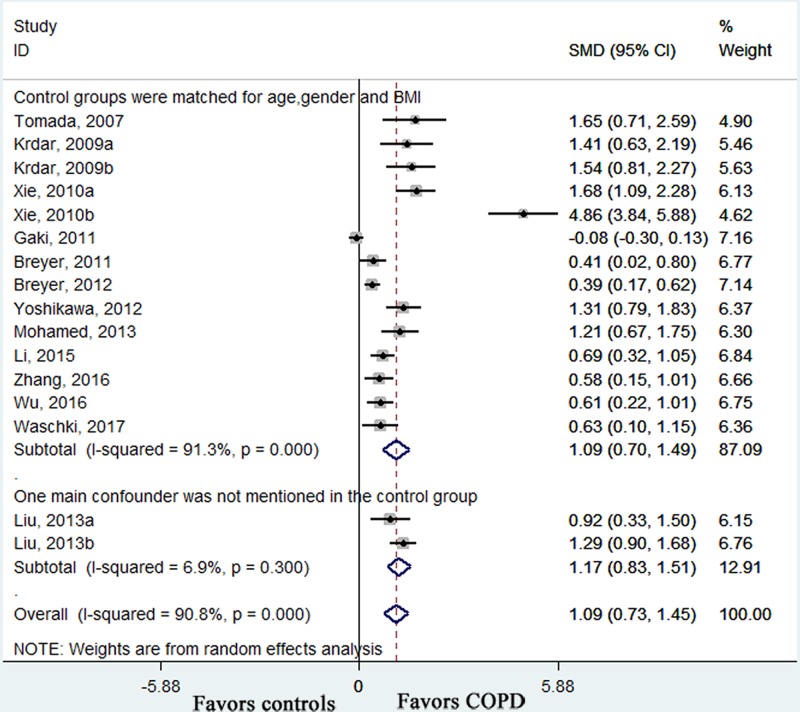
Subgroup analysis based on whether the main confounding factors were controlled Results are expressed as SMD with 95% CI. SMD: standardized mean difference; CI: confidence interval; BMI: body mass index.

All the included studies measured circulating adiponectin levels in COPD patients and healthy control subjects. Pooled effect size showed that the serum adiponectin levels were higher in COPD patients than those in controls (SMD: 1.09, 95% CI [0.73–1.45], *P* < 0.001; *I*^2^ = 90.8%, *P* < 0.001; Figure 2). In the subgroup analyses by disease period, there were similar results in stable COPD patients (SMD: 0.77, 95% CI [0.47–1.07], *P* < 0.001; *I*^2^ = 83.9%, *P* < 0.001; Figure 2), AECOPD patients (SMD: 2.51, 95% CI [0.71–4.30], *P* = 0.006; *I*^2^ = 95.2%, *P* < 0.001; Figure 2) and mixed COPD patients (SMD: 1.21, 95% CI [0.67–1.75], *P* < 0.001; Figure 2). Furthermore, the serum adiponectin levels were higher in AECOPD patients than those in stable COPD patients (SMD: 1.06, 95% CI [0.13–1.99], *P* = 0.026; *I*^2^ = 93%, *P* < 0.001; Figure 3). In order to weaken the influence of confounding variables on the concentrations of adiponectin, subgroup analyses were conducted in those studies which controlled the all main confounding factors (age, gender and BMI). It showed that in that subgroup, the circulating adiponectin levels were still higher in COPD patients than those in controls who were matched for important potential confounders (SMD: 1.09, 95% CI [0.70–1.49], *P* < 0.001; *I*^2^ = 91.3%, *P* < 0.001; Figure 4). The overall results and subgroup analyses in this meta-analysis are presented in Table 3.

**Table 3:** The overall results and subgroup analyses in this meta-analysis

Results	Results	SMDs	95% CI	*P*	*Z*
Overall results	COPD vs Controls	1.09	0.73, 1.45	<0.001	5.96
Subgroup analysis	Stable COPD vs Controls	0.77	0.47, 1.07	<0.001	5.08
	AECOPD vs Controls	2.51	0.71, 4.30	0.006	2.74
	Mixed vs Controls	1.21	0.67, 1.75	<0.001	4.38
	AECOPD vs stable COPD	1.06	0.13, 1.99	0.026	2.23
All main confounding factors were matched	COPD vs Controls	1.09	0.70, 1.49	<0.001	5.45

Abbreviations: 95% CI, 95% confidence interval; AECOPD: acute exacerbation stage of chronic obstructive pulmonary disease; COPD: chronic obstructive pulmonary disease; SMD, standard mean difference.

### Sensitivity analysis

**Figure 5:**
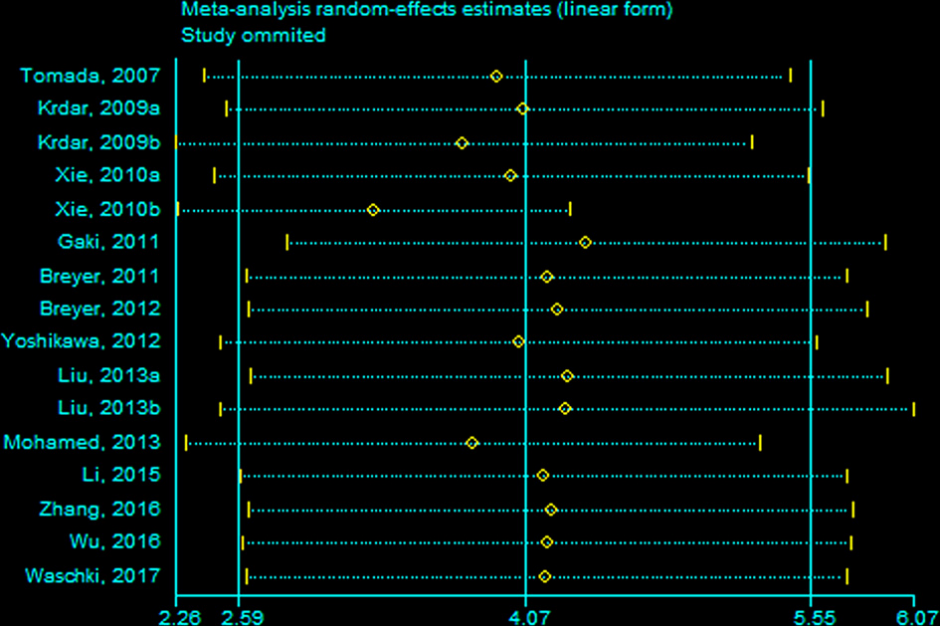
Sensitivity analyses by omitting each study

We performed sensitivity analyses for statistically significant results. In the overall studies, the observed significant results were not materially altered after we sequentially excluded each study (Figure 5).

### Publication bias

**Figure 6:**
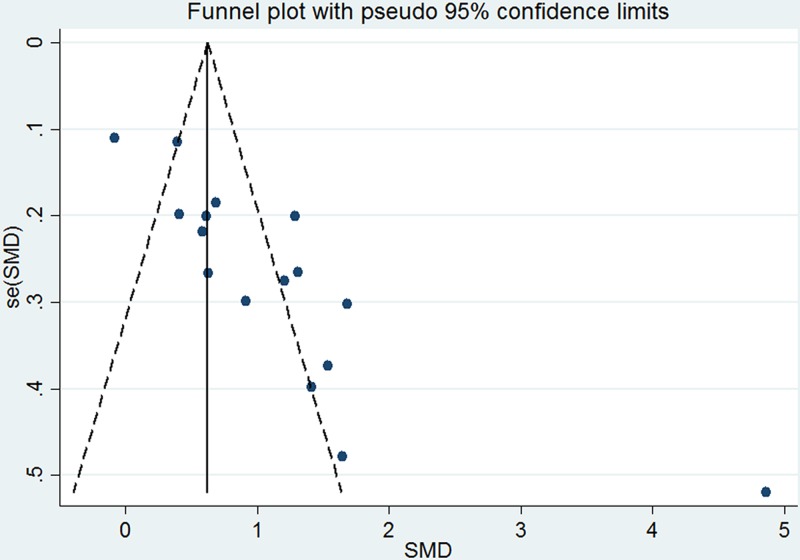
Funnel plots for evaluation of publication bias in the included studies on the association between serum adiponectin concentrations and COPD COPD, chronic obstructive pulmonary disease; SMD, standard mean difference.

Publication biases in the overall meta-analysis were suggested both in the Egger’s test (*P* < 0.001) and Begg’s test (*P* < 0.001). The shape of the funnel plot was asymmetrical (Figure 6).

## Discussion

Adiponectin, a 244-amino-acid-long polypeptide, is a protein hormone involved in a wide variety of physiological processes, such as energy metabolism and inflammation. Dysregulated production of adiponectin can contribute to the pathogenesis of the low-grade systemic inflammation in metabolic disease [28]. However, the potential association between adiponectin and COPD remains controversial. Therefore, we conducted this comprehensive meta-analysis that included all the available data to evaluate the relationship of serum adiponectin levels and COPD. Our findings provide convincing evidence that serum concentrations of adiponectin were higher in COPD patients than in healthy controls. This result of the pooled analysis is in accordance with most findings of previous studies [15–17,19,20,23–25,27].

Serum adiponectin levels correlated inversely with BMI in patients with COPD [20,22,23,27], which indicates that the adipose tissue may be an important influence factor to the serum adiponectin level in COPD. So in this meta-analysis, in order to minimize the effect of BMI, we only included those studies with no significant differences in BMI between the COPD group and control group. It turned out that the serum concentrations of adiponectin were higher in COPD patients than in BMI-matched controls. This result suggests that the elevation of serum adiponectin level may be caused by other pathophysiologic mechanisms besides BMI in COPD. In the subgroup analyses of our study, the circulating adiponectin levels were still higher in COPD patients than those in controls who were matched for important potential confounders (age, gender and BMI), indicating the circulating adiponectin levels can be elevated independently of these confounders in COPD patients.

The circulating adiponectin levels positively correlate with the percentage of predicted residual volume (RV) [23] and dyspnea score [27] but not forced expiratory volume in 1 second (FEV_1_) [23]. These results seem that hyperinflation, not flow limitation, may contribute to the elevation of circulating adiponectin levels in COPD. However, other studies do not reveal similar associations between serum adiponectin levels and other parameters of pulmonary function in COPD patients [19,25,26]. Therefore, the association between the adiponectin and severity of airway obstruction in COPD remains inconclusive.

Moreover, circulating adiponectin level is associated with tumor necrosis factor-α (TNF-α) level [19,23,25], C-reactive protein (CRP) level [20,22], interleukin-8 (IL-8) level [25] and interleukin-6 (IL-6) level [25] in COPD patients. These results reveal elevated adiponectin may play a role in the systemic inflammation in COPD. Meanwhile, adiponectin has various protective anti-inflammatory effects, which may result in a lower risk for the development of atherosclerosis [29]. So, serum adiponectin levels may link with not only the pathophysiology but also the comorbidity in COPD.

Additionally, we detected higher adiponectin levels in the exacerbation period compared with those in stable patients, which indicates an augmented inflammatory response. This result is in good agreement with previous reports [24–26]. Higher adiponectin levels of AECOPD patients can be interpreted as an attempt to overcome the effects of proinflammatory cytokines such as TNF-α, IL-6 and CRP.

The limitations of this meta-analysis should be taken into account. First, although control subjects were matched for BMI, other impact of plausible confounding factors such as pulmonary function and disease duration that could influence the concentration of adiponectin were not possible for us to adjust in our study, for lack of the original data in some included studies. This could explain the heterogeneity in our study. It is important to minimize selection biases to decrease the heterogeneity in future studies. Second, some included studies had small sample size and might not have adequate power to explore the real association. Third, unpublished articles and conference abstracts were not included. This could be why the publication bias was detected in our study.

## Conclusions

Despite the limitations of our present study, to the best of our knowledge, the present study is the first meta-analysis to evaluate the relationship between adiponectin and COPD. It showed that serum adiponectin levels were higher in COPD patients than healthy controls, and serum adiponectin levels were higher in AECOPD patients than stable COPD patients. The present study provides a fundamental and comprehensive approach to further understand the mechanism responsible for the pathogenesis and development of COPD. Longitudinal studies with a larger sample including different COPD stages to evaluate the adiponectin level and COPD seem necessary to better identify the role of serum adiponectin.

## Abbreviations

AECOPD: acute exacerbation stage of chronic obstructive pulmonary disease
BMI: body mass index
CI: confidence interval
COPD: chronic obstructive pulmonary disease
CRP: C-reactive protein
FEV_1_: forced expiratory volume in 1 second
GOLD: Global Initiative for Chronic Obstructive Lung Disease
IL-6: interleukin-6
IL-8: interleukin-8
NOS: Newcastle–Ottawa scale
RV: residual volume
SD: standard deviation
SMD: standard mean difference
TNF-α: tumor necrosis factor-α
